# An investigation of slaughter weight and muscle type effects on carcass fatty acid profiles and meat textural characteristics of young Holstein Friesian bulls

**DOI:** 10.1016/j.heliyon.2024.e27316

**Published:** 2024-03-04

**Authors:** Veysel Fatih Ozdemir, Ridvan Kocyigit, Mete Yanar, Recep Aydin, Abdulkerim Diler, Valiollah Palangi, Maximilian Lackner

**Affiliations:** a Department of Animal Science, College of Agriculture, Ataturk University, 25240, Erzurum, Türkiye; b Department of Plant and Animal Sciences, Vocational School of Technical Sciences, Ataturk University, 25240, Erzurum, Türkiye; c Department of Animal Science, Faculty of Agriculture, Ege University, 35100, Izmir, Türkiye; d Department of Industrial Engineering, University of Applied Sciences Technikum Wien, Hoechstaedtplatz 6, 1200, Vienna, Austria

**Keywords:** Slaughter weight, Fatty acid composition, Textural profile analysis, Meat textural attributes, Sensory evaluation

## Abstract

Study objectives included the assessment of carcass fatty acid composition and meat texture characteristics of younger Holstein Friesian bulls. Three experimental groups were formed based on the weights of the 23 young bulls at slaughter: lighter, medium, and heavier. Samples were taken from the Gluteus medius (GM) and Longissimus thoracis muscles 24 h after slaughter. Fatty acid composition, Warner-Bratzler Meat Shear (WBS) measurements, as well as textural profile analysis (TPA) and sensory analysis of the muscle samples were conducted. The fatty acid composition was determined using Thin Layer Chromatography (HPTLC). Polyunsaturated fatty acids and dietary fatty acids give a neutral hypocholesterolemic effect in direct fluorescent antibody (DFA) contents, DFA/OFA (C14:0+C16:0) ratio, hardness, Warner-Bratzler Shear force and also the chews number – which is desirable - before swallowing (NCBS) the meat were significantly decreased with the increasing slaughter weight. Higher slaughter weight resulted in a larger amount of beef with a better panel tenderness score; however, the meat obtained from the LSW group was less healthy considering the fatty acid profile. Additionally, internal fat contained the highest saturated fatty acids concentrations, while subcutaneous fat contained the highest amount of monounsaturated fatty acids. Furthermore, intramuscular fat levels were highest in PUFA and PUFA/SFA ratio. As a result, this study strongly suggests that slaughter weight and anatomical location of fat samples contribute significantly to meat texture characteristics and fatty acid profiles in Holstein Friesian bulls.

## 1 Introduction

Since pure beef cattle only make up a small portion of the all population in many European countries, beef production is largely derived from dairy breeds, especially Holstein Friesian breeds. Attempts to improve Holstein Friesian cows' dairy characteristics have led to carcasses of low quality. Carcasses are generally used as raw materials for processing meat products since they are less valuable and cheaper [1].

Factors affecting quality, acceptability, physicochemical and textural characteristics, and fatty acid contents of a carcass are classified into genetic (breed or genotype of animal) as well as non-genetic factors, such as diet, age at slaughter, sex, animal handling, and slaughter weight, etc. Among environmental factors, slaughter weight is the most significant factor influencing beef textural quality [2]. Firmness (toughness or degree of tenderness), cohesiveness, and juiciness are the main textural characteristics of meat. Sensory and instrumental methods are various methods of evaluating meat texture. Instrumental methods are mechanical tests such as Texture Profile Analysis (TPA) and Warner Bratzler shear blades. The TPA gives the textural parameters of tenderness (hardness), springiness, cohesiveness, adhesion, gumminess, and chewiness.

Various aspects of meat textural quality have also been significantly affected by the adipose and muscle tissue fatty acid contents [3]. Firmness/oiliness of adipose tissue is be based on its fatty acid profile. It is possible for carcasses to have different intramuscular fat, internal fat, and subcutaneous fat profiles depending on their slaughter weights, with consequent changes in composition, i.e. ratio of monounsaturated fatty acids (MUFAs), polyunsaturated fatty acids (PUFAs), and saturated fatty acids (SFAs) [4,5].

Beef is a significant source of myristic acid, which is a saturated fatty acid and causes serious health problems in human body. Myristic acid plays a major role in the increase of bad cholesterol (LDL). In addition, saturated fatty acids taken with the diet contributes to the formation of insulin resistance, therefore increasing the tendency to diabetes [6,7]. On the other hand, monounsaturated fatty acids have high-density lipoprotein (HDL cholesterol, good cholesterol) increasing effects and play a role in reducing cardiovascular diseases. The most important long-chain polyunsaturated fatty acids are C18, C20 and C22. These long-chain and polyunsaturated fatty acids have a significant effect on biochemical and physiological changes in the body and are known to be essential for healthy nutrition and the human body [8,9]. Additionally, decrease of saturated fatty acids and total fat intake and rising hypocholesterolemic/hypercholesterolemic ratios, PUFA/SFA in human nutrition are recommended by health organizations since they decrease cholesterol levels, as well as low-density lipoprotein (LDL) loading, thereby protecting heart and vascular health [10].

Holstein Friesian cattle, bred on the elevated plains of Eastern Turkey, demonstrate peculiar physical characteristics and are relatively smaller in body weight and size compared to their European lowland counterparts. Although these cattle are smaller, they sustain their unique traits in this region. The male cattle display remarkable adaptability to the challenging environmental conditions of the eastern region, which encompasses diverse climates. The effects of bull slaughter weight on meat characteristics, such as texture, fatty acid composition, and sensory attributes, are not well-understood. Limited data is available on how slaughter weight impacts the properties of beef, particularly in Holstein Friesian cattle bred in harsh Eastern Turkish climatic and geographic conditions. Therefore, the current study aimed to examine the effects of slaughter weight and muscle type on the organoleptic and textural characteristics of meat from Holstein Friesian bulls. Additionally, the impact of slaughter weight and distinct fat depots on the beef's fatty acid composition was also investigated in this research.

## 2 Materials and methods

### Ethical statement

The research was approved by Chairman of the Ethics and Animal Welfare Committee of Faculty of Agriculture at Atatürk University (Approval number:88656144-000.E)

### 2.1 Animals and experimental design

The research was performed at the Food and Livestock Application and Research Center at Ataturk University, Erzurum, Eastern Türkiye. The animal material of the research consisted of 23 of young Holstein Friesian bulls. Group feeding was practiced for 240 days during fattening with the same total mixed ration (TMR). In addition to dry hay, the animals receive 70% concentrate *ad libitum*. The chemical compositions of the feeds are presented in Table 1.

**Table 1:** Chemical compositions of the feed used in this study.

Nutrients, %	Concentrate	Dry hay
Crude protein	17.1	9.2
Dry matter	88.6	92.7
Crude ash	8.7	8.8
Acid detergent fiber	20.4	38.2
Neutral detergent fiber	39.8	64.7
Ether extract	2.2	2.9

After the fattening period was completed, they were transferred to the Meat and Milk Board abattoir about 10 km from the Research Center. Before slaughtering the young bulls, they were allotted into three experimental groups according to their slaughter weights called as lighter (n: 7, ranged from 415 to 540 and average: 476.6 ± 40.5 kg) (LSW), medium (n: 9, ranged from 529 to 570 and average: 546.9 ± 15.7 kg) (MSW) and heavy (n: 7, ranged from 580 to 665 and average: 607.8 ± 30.5 kg) (HSW) slaughter weight groups. The average slaughter age of the young bulls included in this study was 21.5 ± 0.6 months. Slaughtering and post-slaughter procedure was performed according to the Turkish Standards Institute slaughtering and carcass preparation regulations [11]. Carcasses were chilled in a cold storage room at 4 °C for 24 h.

### 2.2 Samples collection

At 24 h postmortem, muscle pieces were sampled from the *Gluteus medius* (GM) and *Longissimus thoracis* (LT) muscles. An organoleptic and textural analysis of the muscle portions was performed using three pieces of muscle cut perpendicular to the muscle fiber.

At 24 h postmortem, the beef samples were stored in a refrigerator at −20 °C until sensory panel tests were performed as well as texture profile analysis (TPA) and Warner-Bratzler shear force (WBS). Plastic bags were used to store the meat samples before cooking. Their internal temperature was then raised to 70 °C by immersing them in a water bath at 90 °C [12]. To remove cooking drips from cooked samples, a paper towel was placed over them for 5 min. A cooking yield was ratio of the cooked meat weight to the uncooked weight of the meat.

### 2.3 Sensory evaluation

A nine-member test panel was used to evaluate the palatability attributes of LT and GM muscles from carcasses in LSW, MSW, and HSW groups. The cooked beef samples were portioned into pieces of uniform dimensions (about 10 g), and they were served randomly to the nine trained sensory panelists. Distilled water was also provided for the removal of residual flavor notes between sample evaluations. A 9-point descriptive scale was utilized to rate them based on their juiciness, flavor intensity, tenderness, and general acceptability (from 1 = highly unacceptable to 9 = highly desirable). A sensory panel also determined how many chews were taken before swallowing (NCBS).

### 2.4 Textural properties

#### 2.4.1 Warner–Bratzler shear force measurement

WBS analysis was conducted with a Warner-Bratzler Meat Shear (G-R Manufacturing Co. Manhattan, KS, US) using a WBS attachment (crosshead speed of 200 or 250 mm/min. We cooled cooked meat samples to 20 °C to determine the tenderness of each muscle fiber and then removed six round cores at 0.5-inch diameter perpendicular to the longitudinal orientation so the shearing action was perpendicular to the longitudinal orientation. The statistical analysis was based on the average of the six measurements.

#### 2.4.2 Textural profile analysis

In TPA analysis, meat samples that were cooked and cooled to 20 °C were tested on a texture analyzer (CT3 Texture Analyzer, Brookfield Engineering, USA). A 50.8 mm diameter cylindrical probe (TA25/1000, Brookfield Engineering, USA) was used to analyze cylindrical samples (20 mm diameter by 20 mm height). During the test, the pre-test speed was set at 1 mm/s. The actual test speed as well as the post-test speed were set at 2 mm/s, the recovery time was 5s, and the target strain was 50%. As described by Bourne [13], the TPA parameters such as adhesiveness, hardness, cohesiveness, gumminess, springiness, resilience and chewiness were obtained from the force-time plots.

### 2.5 Determination of fatty acid profile

At 24 h post-mortem, samples of internal fat depots (omental fat, channel, and kidney knob) and LT muscle (intramuscular fat) were taken from each carcass for fatty acid analysis. The samples were homogenized for 2 min using a tissue homogenizer at 5000 rpm for 2 min with 4 ml of Sodium dodecyl sulphate (SDS) 10%. Samples were homogenized in an ice-bath. In order to determine fatty acids profiles of the samples, High-Performance Thin Layer Chromatography (HPTLC) was used. The HPTLC analysis were performed at room temperature (25 °C). Fatty acids were identified using the standard in a Supelco 37 FAME mixture (C4–C24) (Bellefonte, PA, USA). An Eppendorf tube containing 1000 ml of meat homogenate was first soaked in an n-hexane: isopropanol (mixed 3:2 by volume) to extract meat lipids. A chromatographic analysis of meat lipids was performed using the upper phase of the centrifuged tubes after vortexing vigorously for 5 min at +4 °C. In order to separate and identify the samples, HPTLC plates measuring 20 × 10 cm were used. Dipalmitoylphosphatidylcholine, 13, dipalmitin, and triolein make up the standard lipid mixture. A micropipette was used to spot 3–6 g of each of the components from standards and 2.5 l of meat lipid extracts on HPTLC plates 2 cm from the bottom. A ratio of 80:20:2 (vol/vol) of n-hexane: diethyl ether: formic acid was used to develop the lipids. By charring at 180 °C for about 10 min, lipid classes were visualized after the plate had been developed with 10% CuSO_4_ (w/v) in 8% H_3_PO_4_ (v/v). An Epson Perfection V700 Photo scanner scanned HPTL chromatograms. The resolution was 600 DPI, the data were recorded as grayscale TIFF images. The software programme TL 120 was used for analysis. We calculated fatty acid profile percentages (g/100 g of total fatty acids) by dividing the total lipid content of the samples by the percentage of each fatty acid class Kaynar et al. [14].

### 2.6 Statistical analysis

To test the normality of the study's data, the Kolmogorov-Smirnov test was used in SPSS statistical program version 20. Statistical analysis was performed SPSS GLM since the data had a normal distribution. Three different mathematical models were formed to analyze the effects of fixed factors SPSS [15]. Data on chemical composition, sensory panel evaluations, color characteristics, and textural characteristics were analyzed by a two-way ANOVA. Since interactions between muscle types and slaughter weights, and between fat types and slaughter weights were not statistically significant in the preliminary analysis, they were excluded from the mathematical models used in the final statistical analysis.

The model (1) used for statistical analysis of data on the fatty acid content was as follows:

(1) Y_ifk_ = μ+a_i_ + b_f_ + e_ifk_

The second mathematical model (2) used for ANOVA of data on the sensory panel evaluation, meat textural characteristics and proximate analysis was as follows:

(2) Y_ilk_ = μ+a_i_ + c_l_ + e_ilk_

Where, Y represents each of the observations. μ is the least squares means while a, b, c respectively corresponded to the fixed effects of the slaughter weight (i = 1, lighter; i = 2, medium; i = 3, heavier), fat types (1 = internal fat; 2 = subcutaneous fat) and muscle types (1 = LD; 2 = GM). The random residual effect was indicated by e. Comparison of the subclass means that are significantly different each other was performed by using Duncan's Multiple Range Test.

## 3 Results

Results concerning flavor intensity, tenderness, general acceptability, NCBS and cooking yield are presented in Table 2. NCBS and the all sensory panel scores were significantly affected by muscle types. In contrast, slaughter weights were the only significant sources of variation in the tenderness scores, WBS, and NCBS. Neither slaughter weights nor muscle types significantly affected the cooking yield of GM and LT muscles of Holstein Friesian Bulls in LSW, MSW, and HSW groups.

**Table 2:** Sensory attributes and cooking yield (least squares-means ± standard errors).

Sensory Attributes	Slaughter Weights				Muscle Types		
	LSW (n = 7)	MSW (n = 9)	HSW (n = 7)		GM (n = 23)	LD (n = 23)	
Tenderness	5.29 ± 0.23^b^	5.79 ± 0.21^ab^	6.06 ± 0.23^a^	*	5.45 ± 0.18^a^	5.99 ± 0.18^b^	*
Juiciness	5.58 ± 0.18	5.48 ± 0.16	5.89 ± 0.18	ns	5.32 ± 0.14^a^	5.98 ± 0.14^b^	**
Acceptability	6.16 ± 0.17	6.03 ± 0.15	6.49 ± 0.17	ns	5.96 ± 0.13^a^	6.49 ± 0.13^b^	**
Palatability	5.88 ± 0.18	5.64 ± 0.16	6.18 ± 0.18	ns	5.65 ± 0.14^a^	6.15 ± 0.14^b^	**
Flavor Intensity	5.95 ± 0.18	5.66 ± 0.16	5.98 ± 0.18	ns	5.68 ± 0.14	6.04 ± 0.14	*
NCBS	33.17 ± 1.42^a^	32.82 ± 1.25^ab^	29.06 ± 1.42^b^	*	33.08 ± 1.12	30.29 ± 1.12	*
Cooking Yield	68.48 ± 1.12	69.18 ± 0.98	69.83 ± 1.12	ns	69.53 ± 0.87	68.80 ± 0.87	ns

LSW: Lighter Slaughter Weight, MSW: Medium Slaughter Weight, HSW: Heavy Slaughter Weight, NCBS: Chews number before swallowing, GM: *Gluteus medius*, LD: *Longissimus dorsi*, ^a, b^: Means in rows with dissimilar superscript differ statistically, *: *P* < 0.05, **: *P* < 0.01, ns: non-sig.

The means and standard errors for meat texture parameters a Warner-Bratzler Shear values are tabulated in Table 3. While LSW, MSW, and HSW groups resulted in significant variation in WBS values (*P* < 0.01) and hardness (*P* < 0.05), the chewiness, gumminess and hardness of texture characteristics were affected statistically significant (*P* < 0.01) by muscle types.

**Table 3:** Meat texture parameters and Warner-Bratzler Shear value (least squares-means with standard errors).

Texture Parameters	Slaughter Weights				Muscle Types		
	LSW (n = 7)	MSW (n = 9)	HSW (n = 7)		GM (n = 23)	LD (n = 23)	
Adhesiveness	0.132 ± 0.06	0.112 ± 0.05	0.213 ± 0.06	ns	0.106 ± 0.05	0.200 ± 0.05	ns
Hardness (N)	89.18 ± 4.63^a^	81.85 ± 4.08^ab^	73.91 ± 4.63^b^	*	97.76 ± 3.63^a^	65.54 ± 3.63^b^	**
Cohesiveness	0.403 ± 0.01	0.408 ± 0.01	0.414 ± 0.01	ns	0.403 ± 0.01	0.414 ± 0.01	ns
Resilience (N mm)	0.129 ± 0.01	0.129 ± 0.01	1.133 ± 0.01	ns	0.131 ± 0.01	0.130 ± 0.01	ns
Gumminess (N)	35.56 ± 2.11	33.53 ± 1.86	30.59 ± 2.11	ns	39.36 ± 1.66^a^	27.09 ± 1.66 ^b^	**
Springiness (mm)	4.47 ± 0.15	4.55 ± 0.14	4.36 ± 0.15	ns	4.50 ± 0.12	4.42 ± 0.12	ns
Chewiness (N)	155.83 ± 11.13	155.34 ± 9.82	134.21 ± 11.13	ns	176.23 ± 8.72^a^	120.68 ± 8.72^b^	**
WBS (lb)	14.00 ± 0.54^a^	13.67 ± 0.48^a^	11.29 ± 0.54^b^	**	12.87 ± 0.42	13.10 ± 0.42	ns

LSW: Lighter Slaughter Weight, MSW: Medium Slaughter Weight, HSW: Heavy Slaughter Weight; WBS:Warner-Bratzler Shear value, GM: *Gluteus medius,* LD: *Longissimus dorsi*, ^a, b^: Means in rows with dissimilar superscript differ statistically, **: *P* < 0.01, *: *P* < 0.05, ns: non-sig.

The means and standard errors for fatty acid profiles of the LT muscle as well as internal and subcutaneous fats of young bulls in LSW, MSW, and HSW groups are provided in Table 4. Different slaughter weight groups resulted in significant (*P* < 0.05) impact on the PUFA/SFA ratios and PUFA, while the types of fat had significant (*P* < 0.01) effect on the MUFA, UFA, PUFA, SFA as well as PUFA/SFA, UFA/SFA ratios. Slaughter ages and fat types had significant (*P* < 0.01) effects on dietary fatty acids. An undesirable hypercholesterolemic effect was found in humans (C14:0+C16:0) (OFA). Dietary fatty acids were found to give a desired neutral hypocholesterolemic effect in humans (UFA + C18:0) (DFA) contents along with DFA/OFA ratios.

**Table 4:** Fatty acids profile of the LT muscle as well as internal and subcutaneous fats of Holstein bulls slaughtered at three live weights (least-squares means ± standard errors).

	Slaughter Weights				Fat Types			
	LSW	MSW	HSW		Intramuscular fat of LD	Internal Fat	Subcutaneous Fat	
	g/100 g of Fatty Acids							
C14:0	2.039 ± 0.104^b^	2.091 ± 0.092^b^	2.605 ± 0.104^a^	**	1.131 ± 0.100^b^	2.793 ± 0.100^a^	2.811 ± 0.100^a^	**
C14:1	0.671 ± 0.039	0.623 ± 0.035	0.684 ± 0.039	ns	0.684 ± 0.038	0.667 ± 0.038	0.628 ± 0.038	ns
C15:0	0.650 ± 0.043^b^	0.677 ± 0.038^b^	0.887 ± 0.043^a^	**	0.667 ± 0.041^b^	0.825 ± 0.041^a^	0.723 ± 0.041^ab^	*
C15:1	0.490 ± 0.020^ab^	0.477 ± 0.018^b^	0.542 ± 0.020^a^	*	0.500 ± 0.019^ab^	0.543 ± 0.020^a^	0.466 ± 0.019^b^	*
C16:0	23.524 ± 0.336^b^	25.023 ± 0.296^a^	25.839 ± 0.336^a^	**	25.792 ± 0.322^a^	23.191 ± 0.322^b^	25.403 ± 0.322^a^	**
C16:1	2.500 ± 0.221	2.210 ± 0.195	2.314 ± 0.221	ns	2.731 ± 0.212^b^	0.865 ± 0.212^c^	3.423 ± 0.212^a^	**
C17:0	1.248 ± 0.040^ab^	1.213 ± 0.036^b^	1.343 ± 0.040^a^	*	1.143 ± 0.038^b^	1.457 ± 0.039^a^	1.204 ± 0.038^b^	**
C17:1	1.421 ± 0.071^a^	1.292 ± 0.063^ab^	1.124 ± 0.073^b^	*	2.001 ± 0.068^a^	0.998 ± 0.070^b^	0.838 ± 0.068^b^	**
C18:0	25.852 ± 0.554^a^	24.946 ± 0.489^ab^	23.941 ± 0.554^b^	*	22.157 ± 0.531^b^	31.569 ± 0.531^a^	21.013 ± 0.531^b^	**
C18:1n9c	33.797 ± 0.550	34.206 ± 0.485	33.470 ± 0.550	ns	34.978 ± 0.527^b^	29.471 ± 0.527^c^	37.025 ± 0.527^a^	**
C18:1n9t	2.612 ± 0.88	2.652 ± 0.78	2.621 ± 0.90	ns	3.524 ± 0.084^a^	2.460 ± 0.084^b^	1.901 ± 0.084^c^	**
C18:2	3.798 ± 0.116^a^	3.453 ± 0.102^b^	3.460 ± 0.116^b^	*	3.781 ± 0.111^b^	3.792 ± 0.111^a^	3.138 ± 0.111^b^	**
C18:3	0.253 ± 0.015	0.264 ± 0.013	0.227 ± 0.015	ns	0.181 ± 0.014^b^	0.354 ± 0.014^a^	0.209 ± 0.014^b^	**
C20:0	0.751 ± 0.035	0.701 ± 0.031	0.755 ± 0.035	ns	0.668 ± 0.034^b^	0.772 ± 0.034^a^	0.767 ± 0.034^a^	*
C20:1	0.407 ± 0.021	0.429 ± 0.019	0.392 ± 0.021	ns	0.375 ± 0.021^b^	0.333 ± 0.021^b^	0.520 ± 0.021^a^	**
MUFA	41.898 ± 0.572	41.875 ± 0.505	41.021 ± 0.572	ns	44.795 ± 0.548^a^	35.278 ± 0.548^b^	44.720 ± 0.548^a^	**
SFA	54.065 ± 0.567	54.600 ± 0.500	55.370 ± 0.567	ns	51.560 ± 0.543^b^	60.551 ± 0.543^a^	51.924 ± 0.543^b^	**
PUFA	3.989 ± 0.116^a^	3.641 ± 0.102^b^	3.624 ± 0.116^b^	*	3.780 ± 0.111^b^	4.147 ± 0.111^a^	3.327 ± 0.111^c^	**
MUFA/SFA	0.790 ± 0.021	0.785 ± 0.019	0.750 ± 0.021	ns	0.869 ± 0.02^a^	0.584 ± 0.02^b^	0.873 ± 0.02^a^	**
PUFA/SFA	0.074 ± 0.002^a^	0.067 ± 0.002^b^	0.066 ± 0.002^b^	*	0.074 ± 0.002^a^	0.069 ± 0.002^ab^	0.064 ± 0.002^b^	**
UFA	45.887 ± 0.564	45.516 ± 0.498	44.645 ± 0.564	ns	48.575 ± 0.541^a^	39.425 ± 0.541^b^	48.047 ± 0.541^a^	**
UFA/SFA	0.864 ± 0.022	0.851 ± 0.019	0.816 ± 0.022	ns	0.943 ± 0.021^a^	0.652 ± 0.021^b^	0.937 ± 0.021^a^	**
DFA	71.74 ± 0.421^a^	70.462 ± 0.371^b^	68.586 ± 0.421^c^	**	70.733 ± 0.403^a^	70.994 ± 0.403^a^	69.06 ± 0.403^b^	**
OFA	25.563 ± 0.396^a^	27.114 ± 0.349^b^	28.444 ± 0.396^c^	**	26.923 ± 0.379^a^	25.985 ± 0.379^a^	28.214 ± 0.379^b^	**
DFA/OFA	2.820 ± 0.034^a^	2.621 ± 0.047^b^	2.432 ± 0.054^c^	**	2.636 ± 0.051^a^	2.768 ± 0.051^a^	2.469 ± 0.051^b^	**
n-6	4.051 ± 0.117^a^	3.717 ± 0.103^b^	3.687 ± 0.117^b^	*	3.962 ± 0.112^a^	4.146 ± 0.112^a^	3.347 ± 0.112^b^	**
n-9	36.409 ± 0.544	36.859 ± 0.480	36.001 ± 0.544	ns	38.499 ± 0.521^a^	31.928 ± 0.521^b^	38.841 ± 0.521^a^	**

MUFA: monounsaturated acids, SFA: saturated fatty acids, DFA: dietary fatty acids (UFA + C18:0); UFA = sum of PUFA and MUFA; OFA: dietary fatty acids (C14:0+C16:0); DFA/OFA = hypocholesterolemic/hypercholesterolemic ratio; ^a, b, c^: Means in rows with dissimilar superscript differ statistically, *: *P* < 0.05, **: *P* < 0.01, ns: non-sig.

## 4 Discussion

All results in sensory panel except tenderness did not differ significantly for the slaughter weight group, although the juiciness and palatability scores were slightly higher in the HSW group. The most significant textural parameter of meat quality traits is tenderness which has a significant impact on the consumer satisfaction with meat. Tenderness score of bulls in HSW group was respectively 14.6% and 4.7% greater (*P* < 0.05) compared to LSW as well as MSW groups. However, average NCBS values for HSW group 14.1% and 12.9% lower than those for LSW, and MSW groups (*P* < 0.05) respectively. WBS values were also significantly (*P* < 0.01) influenced by the slaughter weights. Mean WBS value of the meat of the bulls in HSW group had lower (*P* < 0.01) shear force than other groups. The results could be attributed to the strong relationship between tenderness and intramuscular fat content, as already suggested by Jeremiah et al. [16]. In addition, our present research has shown that HSW animals tend to have higher levels of intramuscular fat deposition, which may further enhance tenderness. The findings of our study are also in agreement with the results of Węglarz [17], Lucero-Borja et al. [18] who reported that carcasses from heavier animals were more tender than those from lighter ones.

A statistical difference was determined between muscle types and all sensory panel ratings. As determined by tenderness, juiciness, palatability, flavor intensity, and acceptability tests, LT muscle scored higher than GM muscle. Although the changes in myofibrillar proteins are responsible for tenderness, collagen is a significant part of connective tissue of beef, and the amount of collagen can be affected by body weight. As the connective tissue content in the muscles increases, the meat tends to be tougher. The total amount of collagen varies depending on the position and function of the muscles in the carcass. As a general rule, muscles used for movement contain higher collagen content than muscles used for support or very light movements [19].

The TPA of the meats focused on assessing the differences in tenderness or toughness of the meat. The differences among the slaughter weights in all parameters of the texture analysis except for hardness were statistically insignificant. The meat hardness in HSW group was 20.4% less than those of meat from LSW group. TPA revealed that muscle types (LD and GM) significantly (*P* < 0.01) affected on hardness, gumminess and chewiness, and the values in HSW group were lower than those in LSW group. The study results were in harmony with data of Kopuzlu et al. [20] who stated that different slaughter weights/ages significantly affected meat hardness, gumminess, and chewiness.

It is important to know how meat is composed of fatty acids for human health reasons. Blood levels of low-density lipoprotein cholesterol increase due to SFAs with a chain length of less than 18 carbon atoms, while those with MUFAs and PUFAs decrease. As well as determining the fat tissue firmness, shelf life, color, and flavor of meat, the fatty acid profile also has a crucial effect in defining the quality of the meat [3]. The results of the current study regarding fatty acid composition revealed that there were significant differences between the slaughter weights in terms of Heptadecanoic acid (C17:0) (*P* < 0.05), Pentadecanoic acid (C15:0) and Myristic acid (C14:0), (*P* < 0.01) contents (Table 4). The highest percentages of these fatty acids were obtained from carcasses of the heavy bulls. Likewise, Moreno et al. [4] reported that percentages of C17:0, C15:0, C14:0 increased with an increasing slaughter weight.

SFAs containing stearic acid (C18:0) and palmitic acid (C16:0) had the highest percentages. Likewise, Filipčík et al. [8] noted that stearic acid and palmitic acid were the predominating SFAs in the intramuscular fatty acid pool. HSW group had the highest C18:0 and C16:0 contents while LSW had the lowest, and the differences were statistically significant (*P* < 0.05 and *P* < 0.01). The results are in accordance with data of Moreno et al. [4] and Filipčík et al. [8] who noted an increase in palmitic and stearic acids as slaughter weight increased.

Although epidemiological studies suggested that palmitoleic (C16:1) acid plays a part in cholesterol metabolism and hemostasis, results of the studies regarding its effects on the cardiovascular system were inconsistent [9]. In some studies conducted with diets rich in palmitoleic acid (C16:1), palmitoleic acid was associated with a decrease in LDL cholesterol and plasma triacylglycerols (TG) blood concentrations as well as a significant increase in HDL cholesterol [9,21]. On the other hand, some studies have reported significant correlations between palmitoleic acid and cardiovascular risk factors such as high total cholesterol in the blood, changes in endothelial cells and hypertension. In addition, Chei et al. [22] reported that C16:1 acid content was higher in patients with ischemic heart disease (IHD), but also increased the risk of IHD. Ebbesson et al. [23] pointed out that palmitoleic (C16:1) acid can increase LDL cholesterol level more than palmitic acid (C16:0). The different results in these studies may be due to differences in the subject groups. However, it has been reported that most researchers tend to believe that palmitoleic acid can improve the blood lipid profile [24].

Nevertheless, Shramko et al. [24] found that oleic acid (C18:1n9c) improves blood lipid profiles, maintains healthy body weight, and even reduces insulin resistance and palmitic-SFA-induced mitochondrial dysfunction in neurons and skeletal muscle. Although the effects of slaughter weight on the content of palmitoleic and oleic acid were statistically insignificant in the current research, the highest percentages of these fatty acids were obtained from the LSW group. The results were in harmony with the results of Şahin et al. [5], Hollo et al. [25] and Filipčík et al. [8] also stated a decrease in the C16:1 content with increased slaughter weight in Holstein and Charolaise cattle. However, Nogalski et al. [26] stated that oleic acid levels raised with the increasing of the slaughter weight.

In the current study, differences in the SFA levels of the slaughter weight groups were statistically insignificant, although the percentage of SFA showed a tendency to raise along with increasing of the slaughter weights. The lowest SFA level was determined in LSW group while the greatest SFA content was obtained from HSW group. Insignificant differences determined among the LSW, MSW and HSW groups from the point of percentage of SFA were compatible with findings of Nogalski et al. [26], Hollo et al. [25] (2001) and Mottin et al. [27].

MUFA percentage of the meat from young bulls reared in eastern Turkey slightly decreased with increasing slaughter weight. However, the difference between in MUFA content of the bulls slaughtered at higher weight and lighter weight was found not to be significant. This finding was compatible with results of Mottin et al. [27] who noted insignificant differences among the slaughter weights of Brown Swiss x Nellore crosses in terms of MUFA percentages.

PUFA is deemed to enhance lipid markers by resulting in a decrease in plasma triacylgrycerols as well as ApoB-100 that sequentially diminishes the LDL content. PUFA content of the Holstein bulls in LSW group was significantly (*P* < 0.05) higher than the cattle in other groups, and PUFA percentage decreased as a consequence of raising slaughter weight. Furhermore, Nogalski et al. [1], Moreno et al. [4] and Şahin et al. [5] stated that increasing slaughter weight resulted in a reduction in the percentage of PUFA.

The development of cardiovascular disease was reduced in randomized controlled trials by replacing SFA with PUFA, which led to fewer myocardial infarctions (both fatal and non-fatal combined) and events associated with ischemic heart disease. Therefore, increasing percentage of the PUFA/SFA is regarded desirable for human diets. In the current study, proportion of the PUFA/SFA decreased significantly as the slaughter weight increased (*P* < 0.05). In other words, PUFA/SFA ratio of the young animals in LSW group was 10.4% and 12.1% higher than those of animals in MSW and HSW groups respectively. This result was in harmony with the findings of the researches performed by Węglarz [17], Nogalski et al. [1] and Mottin et al. [27] who reported that the PUFA/SFA ratio lowered as the live weight of the cattle at slaughter time increased. Meanwhile, in present study, the proportion of PUFA/SFA (0.074–0.066) was close to 0.11 that was stated by Enser et al. [28] and lower than that reported by Aldai et al. [29] at 0.45.

Carcasses of the Holstein Friesian bulls in LSW group contained less OFAs (*P* < 0.01) and more (*P* < 0.01) desirable fatty acids (DFAs). Furthermore, percentages of the DFA and the DFA/OFA rate increased significantly (*P* < 0.01) by lowering slaughter weights. In other words, it was found that lighter carcasses of young animals had a more advisable DFA/OFA ratio (the highest value) compared to heavier carcasses.

The percentage of SFA in intramuscular fat was significantly lower (*P* < 0.01) than in internal fat tissues. The finding was compatible with the result of Aldai et al. [29] who indicated that intramuscular fat contained the lowest percentage of SFA compared to subcutaneous fat and intermuscular fat. On the other hand, Sobczuk-Szul et al. [30] indicated the percentages of SFAs from the lowest to the highest as subcutaneous fat (46.8%), intramuscular fat (49.6%), intermuscular fat (57.6%) and visceral fat (59.6%) in crosses of Limousine with Polish Holstein Friesian as well as Polish Holstein Friesian bulls.

Among the three fat tissues examined, intramuscular fat tissue contained the most MUFA compared to internal fat tissue and subcutaneous. However, the reports of the study conducted by Sobczuk-Szul et al. [30] indicated that intramuscular fat had a greater MUFA value (46.2%) than intermuscular fat (38.7%) and visceral fat (36.7%) but was reduced than the subcutaneous fat depot. As opposed to subcutaneous fat depots (39.0%) and intermuscular fat depots (39.9%), Aldai et al. [29] found MUFA values of 33.3% in intramuscular fat tissue.

Dietary fats are usually measured by PUFA/SFA, and 0.45 is considered to be a healthy amount. Lower amounts of PUFA/SFA are not favorable because they may elevate blood cholesterol levels. It is common for ruminant fat to contain PUFA/SFA values that are below the recommended levels [28]. According to the present study, intramuscular fat tissue possessed the highest ratio of PUFAs to SFAs as compared to internal fat tissue and subcutaneous fat tissue. It was found that the PUFA/SFA rate of intramuscular fat was 7.2% higher than that of internal fat and 15.6% higher than that of subcutaneous fat (*P* < 0.01). Furthermore, both Aldai et al. [29] and Sobczuk-Szul et al. [30] pointed out that intramuscular fat had a higher PUFA/SFA ratio than subcutaneous fat, intermuscular fat, and visceral fat.

In DFA and OFA contents and DFA/OF ratios, the type of fat was found to be a relevant source of variation (p < 0.01). Compared with intramuscular fat and internal fat, subcutaneous fat samples contained the highest percentage of OFA. It was observed that internal fat and intramuscular fat had the healthiest DFA/OFA proportion. It was found that the internal fat UFA/SFA rate was the lowest among the different types of fats, with intramuscular fat having the best percentage of UFA/SFA among them. The results from this work were found to be in good agreement with those of Aldai et al. [29].

## 5 Conclusions

A high panel tenderness score and higher OFA content were observed in Holstein Friesian bulls of the heaviest slaughter weight group. On the other hand, an increase in slaughter weight has been accompanied by a significant decrease in PUFA and DFA contents, DFA/OFA ratio, hardness value, Warner-Bratzler Shear force value and NCBS the meat. Although the young bulls in HSW group produced higher amounts of beef with a better panel tenderness score, their meat could be considered less healthy by taking into consideration the fatty acid profile compared to beef from animals in LSW group. While the highest SFA concentrations was found in the internal fat, subcutaneous fat had the highest MUFA content. However, intramuscular fat had a high percentage of PUFA and the highest PUFA/SFA ratio. In conclusion, fatty acid composition and textural features of meat were significantly influenced by different fat depots and weight of Holstein Friesian bulls at slaughter time. Therefore, when evaluating the meat of Holstein-Friesian bulls for human consumption, these two factors should be taken into account.

## Funding

The University of Applied Sciences Technikum Wien is Open Access Funding.

## Ethics committee approval

The study was approved by the local ethics committee of Ataturk University Faculty of Agriculture (number 88656144-000.E).

## Data availability statement

The data will be provided by the authors upon request.

## CRediT authorship contribution statement

**Veysel Fatih Ozdemir:** Writing – original draft, Investigation. **Ridvan Kocyigit:** Methodology, Investigation, Formal analysis. **Mete Yanar:** Investigation, Formal analysis, Data curation. **Recep Aydin:** Validation, Project administration, Methodology. **Abdulkerim Diler:** Supervision, Methodology, Funding acquisition. **Valiollah Palangi:** Writing – review & editing. **Maximilian Lackner:** Writing – review & editing.

## Declaration of competing interest

The authors declare that they have no known competing financial interests or personal relationships that could have appeared to influence the work reported in this paper.
